# Autonoetic Consciousness in Autobiographical Memories after Medial Temporal Lobe Resection

**DOI:** 10.1155/2008/424693

**Published:** 2008-04-11

**Authors:** M. Noulhiane, P. Piolino, D. Hasboun, S. Clemenceau, M. Baulac, S. Samson

**Affiliations:** ^1^Laboratoire de Neurosciences Cognitives et d'Imagerie CérébraleLENA CNRS UPR 640CHU Pitié SalpêtrièreUniversité Pierre et Marie Curie-Paris 6ParisFrance; ^2^Laboratoire de Neuropsychologie et Cognition AuditiveJE 2497Université Charles de Gaule-Lille 3Villeneuve d'AscqFrance; ^3^Laboratoire de Psychologie et Neurosciences CognitivesCNRS FRE 2987Université Paris DescartesParisFrance; ^4^Unité de NeuroradiologieCHU Pitié SalpêtrièreParisFrance; ^5^Unité de NeurochirurgieCHU Pitié SalpêtrièreParisFrance; ^6^Unité d'EpilepsieCHU Pitié SalpêtrièreParisFrance; ^7^Cortex et EpilepsieINSERM 739CHU Pitié SalpêtrièreUniversité Pierre et Marie Curie-Paris 6ParisFrance

**Keywords:** Medial temporal lobe, autobiograhical memory, autonoetic consciousness, Remember/Know paradigm, epilepsy surgery

## Abstract

This study aims to investigate autonoetic consciousness associated with episodic autobiographical memory in patients who had undergone unilateral medial temporal lobe resection for intractable epilepsy. Autonoetic consciousness, defined as the conscious feeling of mentally travelling back in time to relive a specific event, was assessed using the Remember/Know (R/K) paradigm across different time periods as proposed in the autobiographical memory task developed by Piolino et al. (TEMPau task). Results revealed that the two patient groups (left and right temporal resection) gave reduced sense of reliving (R) responses and more familiarity (K) responses than healthy controls. This poor autonoetic consciousness was highlighted when patients were asked to justify their Remember responses by recalling sensory-perceptive, affective or spatiotemporal specific details across all life periods. These results support the bilateral MTL contribution to episodic autobiographical memory covering the entire lifespan, which is consistent with the multiple trace theory of MTL function [7,9]. This study also demonstrates the bilateral involvement of MTL structures in recalling specific details of personal events characterized by autonoetic consciousness.

## 1. Introduction

Since the famous case H.M. [18], it is well known that medial temporal lobe structures (MTL) are involved in episodic memory, which supports the encoding, storage and retrieval of life's events set in a specific spatio-temporal context. Thus, the term episodic memory encompasses not only autobiographical remote memory but also recently learned information. Although most of the studies on temporal lobe epileptic patients as well as on patients who had undergone temporal lobe resection to treat pharmaco-resistant epilepsy are focused on anterograde memory impairments, recent studies have addressed autobiographical memory retrieval. They demonstrated spared personal semantic memory but generally showed deficits of episodic

ISSN 0953-4180/08/$17.00 © 2008 – IOS Press and the authors. All rights reserved autobiographical memory covering the entire lifespan after either right or left MTL lesions [1,5,23,24]. Such findings appear more consistent with the multiple trace theory of MTL function [7,9] rather than the standard model [2,6,8,19]. Nevertheless, previous studies did not examine autonoetic consciousness which gives a subject the conscious feeling of travelling backwards in time to relive the original event ([20] and for review see [21]). This particular state of consciousness during retrieval of information can be assessed with the Remember/Know (R/K) paradigm [3,22]. A 'Remember' response means that the subject recollects a specific event as a re-experiencing of the source of acquisition with details (feelings, perceptions...). By contrast, a 'Know' response corresponds to a feeling of familiarity. Thus, 'Remembering' is associated with autonoetic consciousness whereas 'knowing' is associated with noetic consciousness, which characterizes semantic memory.

The aim of this study was to examine autonoetic consciousness of episodic autobiographical memories in patients who had undergone MTL resection. For this purpose, we used an autobiographical memory task [13] that allows one to obtain a stringent score of episodicity and to differentiate Remember from Know responses [3,22] across different time periods. It also provides the proportion of Remember responses that are precisely justified by asking the participants to recall perceptual, affective and spatio-temporal details related to each specific autobiographical events. According to the current literature which has documented episodic autobiographical memory impairments in these patients, we expected that autonoetic consciousness will be disrupted. If the standard model is valid, this deficit would concern only recent time periods [2,6,8,19]. In contrast, the multiple trace model would predict that all time periods are affected [7,9].

## 2. Methods

### 2.1. Participants

Patients who had undergone left ($n = 12$; age, $34.2 \pm 7.5$ years; educational level, $13.1 \pm 3.5$ years) or right ($n = 10$; age, $34.3 \pm 9.9$ years; educational level, $12 \pm 2.5$ years) MTL resection for the relief of medically intractable epilepsy were tested at La Salpêtrière Hospital. The resection included the amygdala, the hippocampus as well as various amounts of the temporopolar, perirhinal, entorhinal and parahippocampal cortices. To better delineate the resection, we used the MRI volumetric analysis described in detail in a previous article [10]. Table 1 shows the mean volume of the various MTL structures obtained in both hemispheres for the two patient groups. There was no difference in the extent of the removal between patient groups. Patients with evidence of fast-growing tumors or diffuse cerebral damage, intellectual deficiency (full-scale IQ$<$ 75) assessed by the Wechsler Adult Intelligence Scale-Revised [25] or atypical speech representation were excluded. Twenty-two normal control participants matched to the patient groups were also tested ($n = 22$; age, $34 \pm 8.5$ years; educational level, $12.4 \pm 2.7$ years). All participants were right-handed and gave their informed consent in writing before testing in accordance with the Declaration of Helsinki.

Table 1
Mean volumes ($\pm$ S.D.) in mm$^3$ of right and left hippocampus, temporopolar, perirhinal, entorhinal and parahippocampal cortices in patients with right (RTR) or left (LTR) temporal lobe resection

| Cortices | Right hemisphere | Left hemisphere |
|---|---|---|
| Temporopolar | | |
| RTR group | $565 \pm 376$ | $2911 \pm 186$ |
| LTR group | $3111 \pm 280$ | $104 \pm 347$ |
| Perirhinal | | |
| RTR group | $612 \pm 244$ | $2160 \pm 204$ |
| LTR group | $2561 \pm 214$ | $504 \pm 144$ |
| Entorhinal | | |
| RTR group | $404 \pm 156$ | $1498 \pm 57$ |
| LTR group | $1571 \pm 69$ | $366 \pm 73$ |
| Parahippocampal | | |
| RTR group | $1649 \pm 164$ | $2269 \pm 171$ |
| LTR group | $2165 \pm 108$ | $1479 \pm 136$ |
| Hippocampus | | |
| RTR group | $224 \pm 77$ | $3361 \pm 278$ |
| LTR group | $4063 \pm 267$ | $327 \pm 50$ |

### 2.2. Material

Autobiographical memory was assessed with a controlled autobiographical procedure [13], for details about procedure and scoring, see [14,15]) specially designed to measure episodic memories (i.e. unique, specific in time and space, and detailed) from the entire life span across four time periods (P1: 0–17 years old, P2: 18–30 years old, P3: the last 5 years [except the last 12 months], and P4: the last 12 months). The ability to mentally travel back through time and re-experience the source of acquisition, i.e. autonoetic consciousness, was assessed via the 'Remember/Know' paradigm [3, 22]. Moreover, the participant could indicate a Guess response when their response was doubtful. In this study, we focused our interest on the mean proportion of Remember (R) and Know (K) responses rather than Guess responses. The investigator also checked if the subjects were able to *justify* each of their R judgments (justified R responses) in terms of specificity and details. The R, K and justified R responses were expressed as the mean percentage obtained on all periods. For the justified R responses, we also computed the mean percentage according to the remote (by combining P1 and P2) and recent (by combining P3 and P4) periods. A retest was conducted (15 $\pm$ 2 days after the initial task) to check each memory by using a cued recall task which consisted of asking the subjects to recall in random order the content and spatio-temporal situation of each memory. In the three groups of participants, all the memories that were provided at the test were also given at the retest. Two independent experts (MN and PP) rated each memory until a consensus was reached.

## 3. Results

As the data were not normally distributed, neuropsychological scores were compared to those of control participants using non-parametric analyses. Results of the analyses performed on the mean percentage of R and K responses on all time periods revealed a significant effect of Group on R responses ($p < 0.001$) and on K responses ($p < 0.001$). Both RTR and LTR patients gave significantly fewer R responses (RTR: 84%, LTR: 79%) and more K ones (RTR: 10%, LTR: 13%) than controls (R responses: 93%, K responses: 4%). There was no significant difference between RTR and LTR patients. The proportion of justified R responses was lower in the RTR (71%) and LTR (72%) groups than in the control group (92%). This result suggests that patients exhibited poorer capacities than control participants to justify their R responses with sensory and contextual details about the learning context. Therefore, we investigated the percentage of justified R responses according to Group and Period (remote vs recent). LTR and RTR patients were impaired in comparison to the control group across remote and recent periods (*all* $p < 0.05$), without significant differences between the two patient groups. Examination of performance at different periods within each group showed that performance decreased gradually with remoteness in the controls ($p < 0.01$). Although this effect occurred in the two patient groups, it did not reach statistical significance (for details, see Fig. 1).

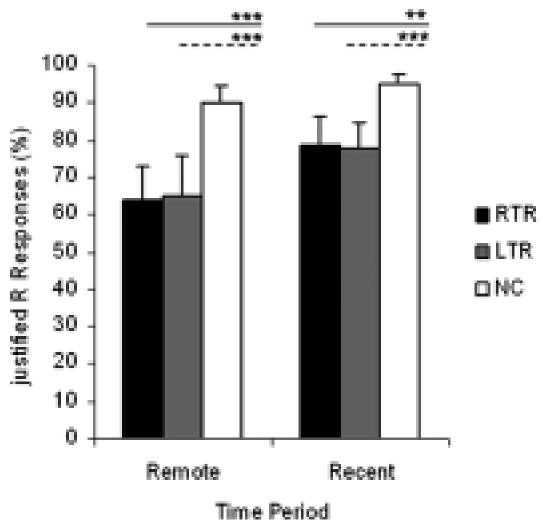

Fig. 1. Percentage of justified R responses according to remote (P1 and P2) and recent (P3 and P4) time periods in patients with right (RTR, $n = 10$) or left (LTR, $n = 12$) temporal lobe resection and normal control participants (NC, $n = 22$). Planned comparisons to illustrate the effect of Group for each period are indicated in the Figure (**$p < 0.01$, ***$p < 0.001$) and the effect of Period for each group are the following: LTR: ns; RTR: ns; NC: Recent > Remote, $p < 0.01$. (Bars indicate the standard errors of the mean).

## 4. Discussion

The aim of this study was to examine autobiographical memory in patients following unilateral MTL resection in order to examine the autonoetic consciousness of personal events. Overall, both LTR and RTR patient groups showed an impairment of episodic autobiographical memory but preservation of personal semantic memory (know responses) which is in agreement with the existing literature [1,5,23,24]. In addition, the two patient groups produced less Remember responses than controls, and had difficulties in justifying their responses across all time periods. This suggests that the patients had significant difficulty recollecting contextual details of encoding and mentally travelling in time. These findings indicate that autonoetic consciousness is altered in patients with unilateral MTL resection. In healthy participants, autonoetic consciousness decreased as a function of the remoteness of events, confirming previous reports [14,15]. However, this effect was not observed in patient groups since they were impaired across all life periods. Our results point to the bilateral involvement of the MTL in re-experiencing either phenomenological or spatio-temporal features of unique autobiographical events across all time periods. Accordingly, some neuroimaging studies have detected bilateral MTL activation when subjects are engaged in the retrieval of specific autobiographical memories [4, 12,16,17,20]. Another study [11] will explore the relationship between episodic autobiographical memory measures and the volume of various MTL regions to better understand the contribution of each of these structures to autonoetic conciousness. Finally, the impairment of autonoetic consciousness extends to the most remote time periods in early childhood. This finding supports the multiple trace theory, which suggests that various MTL regions permanently contribute to the memory of remote as well as recent episodic autobiographical events [7,9].

## Acknowledgements

This work was supported by the French League Against Epilepsy (Novartis to M.N.) and the Regional Council of Nord Pas de Calais Picardie (Ph.D. fellowship to M.N.). We wish to thank Aimee Baird for reviewing the English style.
